# Synergistic interaction of renewable nipagin and eugenol for aromatic copoly(ether ester) materials with desired performance

**DOI:** 10.1038/s41598-021-03614-z

**Published:** 2021-12-16

**Authors:** Keling Hu, Huachao Sui, Dongping Zhao

**Affiliations:** 1 School of Materials Science and Engineering, Tiangong University, Tianjin, 300387 People’s Republic of China; 2 School and Hospital of Stomatology, Tianjin Medical University, Tianjin, 300070 People’s Republic of China

**Keywords:** Chemistry, Materials science

## Abstract

Naturally occurring nipagin and eugenol were used as the collaborative starting materials for poly(ether ester) polymers. In this study, two series of nipagin and eugenol-derived copoly(ether ester)s, PHN1_1−x_E1_x_ and PHN1_1−x_E2_x_ (x = 0%, 5%, 10%, 15%, 20%), were prepared with renewable 1,6-hexanediol as a comonomer. The nipagin-derived component acts as the renewable surrogate of petroleum-based dimethyl terephthalate (DMT), while the eugenol-derived component acts as the cooperative property modifier of parent homopoly(ether ester) PHN1. 1,6-Hexanediol was chosen as the spacer because of its renewability, high boiling point, and short chain to enhance the glass transition temperatures (*T*_g_s) of materials. The molecular weights and chemical structures were confirmed by gel permeation chromatograph (GPC), NMR and FTIR spectroscopies. Thermal and crystalline properties were studied by thermal gravimetric analysis (TGA), differential scanning calorimetric (DSC) and wide-angle X-ray diffraction (WXRD). The tensile assays were conducted to evaluate the mechanical properties. The results suggested that properties of this kind of poly(ether ester)s could be finely tuned by the relative content of two components for the desired applications (elastomer, rubbery) suitable for different scenarios from polyethylene glycol terephthalate (PET) and polybutylene terephthalate (PBT).

## Introduction

Currently, great majority of plastic consumed globally is produced from fossil resources. This situation brings about the gradual depletion of non-renewable materials, as well as increasingly severe environmental concerns^1–6^. The reality prompts us to find bio-based substitutes to petroleum-based resources from the view of sustainable development^7–11^. PET and PBT are representative examples of widely used engineering plastics, but they are mainly produced from petroleum-based dimethyl terephthalate (DMT). Due to the huge consumption, finding the renewable surrogates for DMT without sacrificing the thermal stability and mechanical strength of materials is urgent.

Nipagin and eugenol are two naturally occurring building blocks, which exist in campanulaceae plant and clove, respectively^12,13^. These two starting materials have been found widespread applications in fields of liquid crystalline material^14,15^, epoxy resin^16–18^, benzoxazine^19,20^, coating^21,22^, and Li–S battery^23^. Because of the multifunctionality (phenol and vinyl) and renewability of nipagin and eugenol, based on them our group has been dedicated to polyester materials, whose thermal, crystalline, mechanical and degradable properties have been comprehensively investigated^24,25^. The influence of monomer structure on final properties was also studied^26^. However, the comprehensive performance of obtained polyesters can still be improvable, regardless of the renewability of starting materials. Either the glass transition temperatures (*T*_g_s) were not high enough, or the mechanical tough and strength was not perfect. Consequently, further optimizing the performance of nipagin and eugenol-based polymer materials is particularly important for the development and utilization of such materials.

Considering the structural difference of nipagin and eugenol, the symmetrical nipagin units can endow the polymers with high crystallinity and mechanical strength, but poor flexibility and ductility^27,28^. In contrast, eugenol-derived unit is highly asymmetrical one, which endows the polymers low crystallinity and poor mechanical strength, while their flexibility and ductility are good^24^. Taking advantages of the complementary features of these two building blocks through rational molecular design may give the polymeric materials synergistic effect and desired final properties.

In this work, three nipagin and eugenol-derived diester monomers were firstly synthesized (Supplementary information, Fig. S1-S8), then melt polycondensation between diester monomers and 1,6-hexanediol were carried out. 1,6-Hexanediol was chosen because of its renewability and high boiling point (250 °C, 1 atm) compared with short-chain ethylene glycol (196 °C, 1 atm) and 1,4-butanediol (228 °C, 1 atm)^29–32^, the low boiling points of which can lead to volatilize easily during the polycondensation stage and be difficult to control the stoichiometric ratio between diester and diol precisely, and thus caused low molecular weights. Meanwhile, due to the chain length of 1,6-hexanediol is shorter than that of 1,10-decanediol, the *T*_g_s of obtained polymers were enhanced obviously compared with our previously synthesized polyesters with 1,10-decanediol as the spacer^26^. The high *T*_g_ values can make polymer materials applied in a wide temperature range. Synergistic effect between these two structurally distinctive components (nipagin and eugenol) endows the materials with desired performance, which can be modulated for a broad range of applications (elastomer, rubbery) suitable for a variety of scenarios.

## Results and discussion

### Synthesis and structures of the copoly(ether ester)s

**Figure 1:**
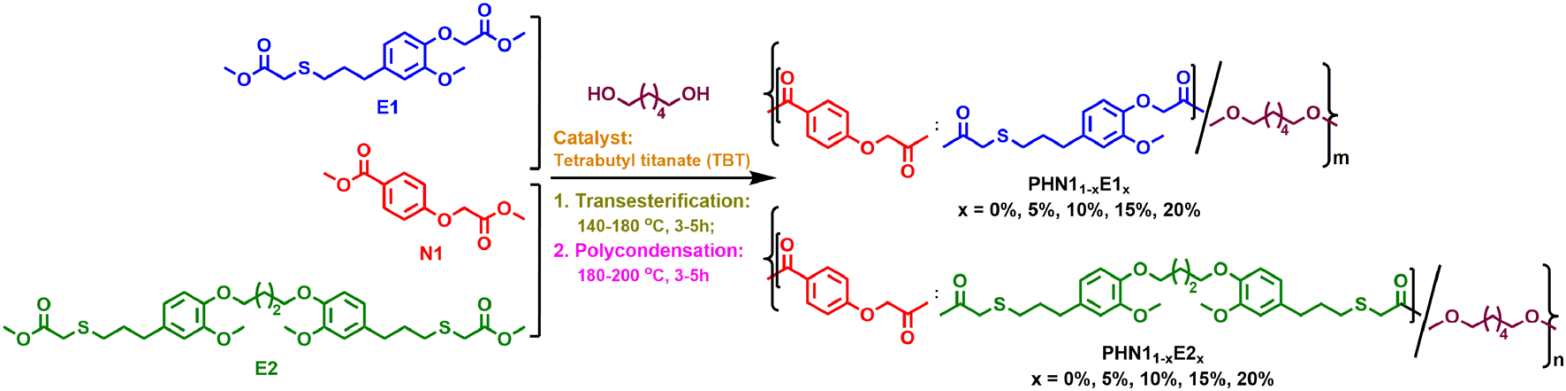
Synthetic procedures for the preparation of nipagin and eugenol-based poly(ether ester)s. The polymerization was performed in a 25 mL Schleck round-bottom flask equipped with a nitrogen inlet, a vacuum distillation outlet, and a stirrer bar. The catalyst, polymerization temperature and times are indicated in the figure.

PHN1_1−x_E1_x_ and PHN1_1−x_E2_x_ copoly(ether ester)s were synthesized by a melt polycondensation method according to the predetermined composition ratios of nipagin and eugenol-derived dimethyl ester monomers. The polymerization routes are shown in Fig. 1 and the results are summarized in Table 1. The GPC traces are depicted in Supplementary Fig. S9-S10.

**Table 1:** Molar composition, molecular weight, polydispersity, isolated yield and appearance of the synthesized poly(ether ester)s.

Entry	Poly(ether ester)	Yield (%)^c^	Molar composition				Molecular weight^b^			Isolated appearance ^c^
			Feed ratio		Final product^a^					
			*X*_N1_	*X*_E_	*X*_N1_	*X*_E_	*M*_n_	*M*_w_	*Ð*	
1	PHN1	92%	100	0	100	0	14,900	26,300	1.8	White powder
2	PHN1_95%_E1_5%_	87%	95	5	94.3	5.7	13,400	23,600	1.8	Light yellow powder
3	PHN1_90%_E1_10%_	88%	90	10	90.4	9.6	10,200	16,900	1.7	Light yellow powder
4	PHN1_85%_E1_15%_	86%	85	15	84.7	15.3	10,200	17,200	1.7	Light yellow powder
5	PHN1_80%_E1_20%_	85%	80	20	80.3	19.7	13,900	24,000	1.7	Light yellow semi-solid
6	PHN1_95%_E2_5%_	87%	95	5	94.3	5.7	15,100	26,800	1.8	Light yellow powder
7	PHN1_90%_E2_10%_	85%	90	10	89.3	10.7	13,200	22,400	1.7	Light yellow powder
8	PHN1_85%_E2_15%_	86%	85	15	84.0	16.0	13,200	23,000	1.7	Light yellow semi-solid
9	PHN1_80%_E2_20%_	82%	80	20	78.7	21.3	11,400	19,900	1.7	Light yellow semi-solid

^a^Molar composition in final products determined by the integration of ^1^H NMR spectra.

^b^Polystyrene (PS) calibrated gel permeation chromatography (GPC) values with tetrahydrofuran (THF) eluent.

^c^After purification by precipitating from an excess amount of methanol and dried in vacuum overnight.

The high yields suggest the high reactivity of the two kinds of monomers. The consistency of nipagin and eugenol-derived units in the feed and final product demonstrates that nipagin and eugenol-based diester monomers have similar reactivity and excellent compatibility. Copoly(ether ester)s with *M*_n_ above 10^4^ were successfully obtained, and polydispersity (*Ɖ* = *M*_n_*/ M*_w_) values are found to be fixed at about 1.7 regardless of the feed ratios, which also suggest the similar reactivity of diester monomers, where *M*_n_ and *M*_w_ are the number- and weight-average molecular weights, respectively. The apparent appearance of final products is closely related with the feed ratios. That is, with the gradual increase of eugenol-based units, the physical form of final product changes from white powder for PHN1, transforms into yellow powder for PHN1_95%_E1(2)_5%_, PHN1_90%_E1(2)_10%_, and PHN1_85%_E1_15%_, finally becomes light yellow semi-solid for PHN1_80%_E1_20%_, PHN1_85%_E2_15%_ and PHN1_80%_E2_20%_. This phenomenon indicates that eugenol-derived units are easier to be oxidized than nipagin-derived units.

**Figure 2:**
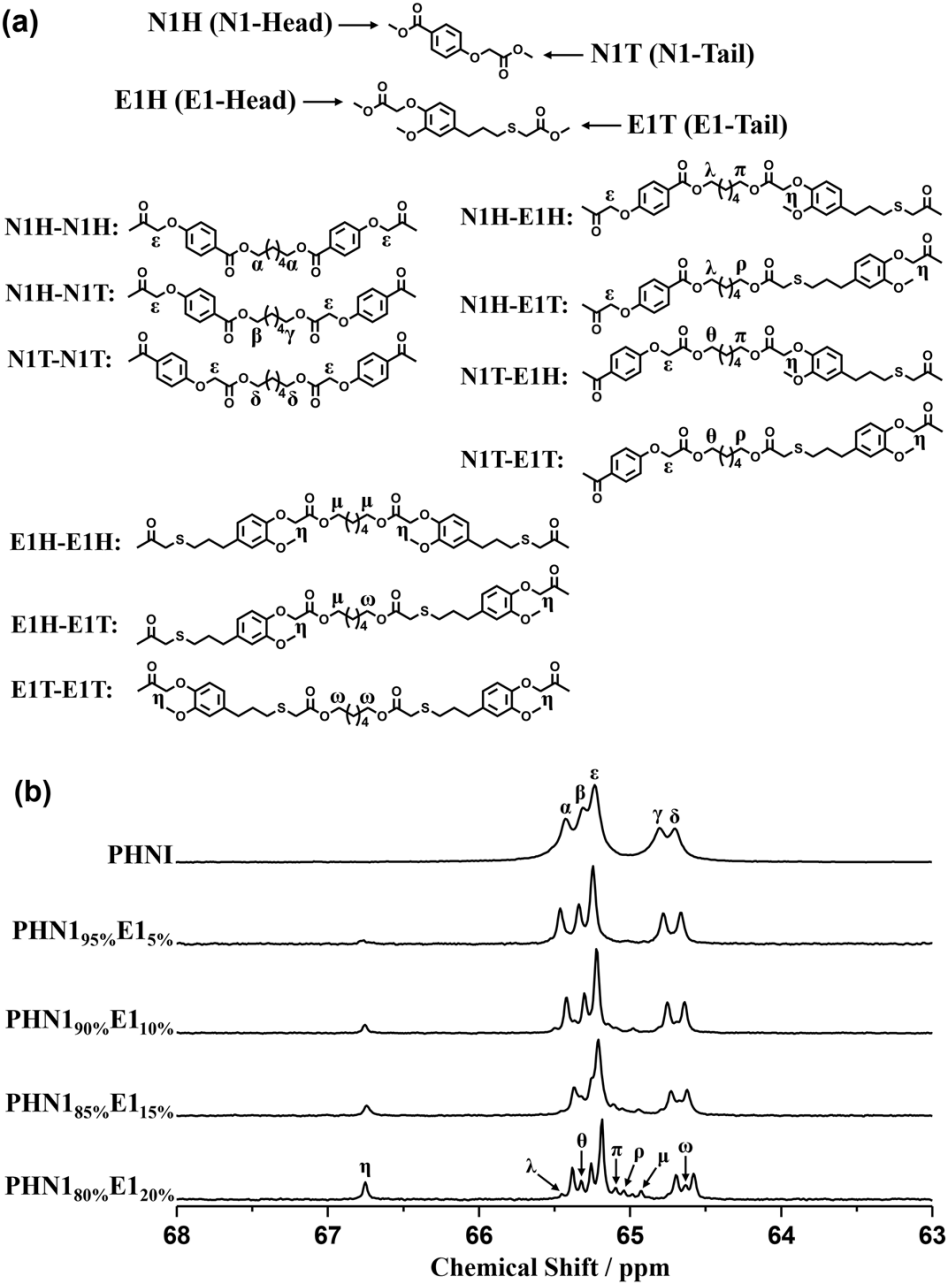
The splitting situations (**b**) of different carbons under magnetically non-equivalent environment along polymer chains for PHN1_1−x_E1_x_ with the indications of dyads to which they are assigned (**a**). N1H and N1T indicate the *head* and *tail* sides of N1 unit, while E1H and E1T indicate the *head* and *tail* sides of E1 unit. The ^13^C NMR spectra were recorded in deuterated chloroform (CDCl_3_) at 25 °C on a Bruker AVANCE III NMR spectrometer operating at 100.6 MHz.

As the structural information of polymer chains are closely related to thermal, crystalline and mechanical properties of materials, chemical structures of the copoly(ether ester)s were confirmed by ^1^H NMR (see Supplementary Fig. S11-S12), ^13^C NMR (see Supplementary Fig. S13-S14) and FTIR spectroscopy (see Supplementary Fig. S15-S16). Furthermore, their chemical microstructures were studied by the quantitative ^13^C NMR spectra taking advantage of the sensitiveness of magnetically different carbon atoms present in backbones towards sequence distributions at the dyed level^33^. In the present study, the methylene carbons adjacent to the alcohol-oxygens were well resolved in the ^13^C NMR spectra due to the asymmetrical feature of N1 and E1. They exhibited the difference of *head* and *tail* when incorporated into the polymer chains during copolymerizing. However, when N1 was copolymerized with E2, N1 displayed the difference of *head* and *tail* while the two ester groups in E2 were equivalent. The possible sequence distributions and splitting situation of methylene carbons in ^13^C NMR spectra were depicted in Fig. 2 and Supplementary Fig. S17, from which we can observe that the signal intensities depend closely on the feed ratios of copoly(ether ester)s. Due to short chain of 1,6-hexanediol, the splitting of methylene carbons adjacent to the alcohol-oxygens was very sensitive to sequence distributions as shown in the case of PHN1_80%_E1_20%_ (Fig. 2b). This analysis suggested that N1 and E1(E2) were incorporated into the copoly(ether ester) chains in an arbitrary manner. Hence, the nipagin and eugenol-derived copoly(ether ester)s have completely random microstructures in polymer mainchains. This randomness may have vital influence on thermal stability, crystallinity, and mechanical strength.

### Thermal properties

**Figure 3:**
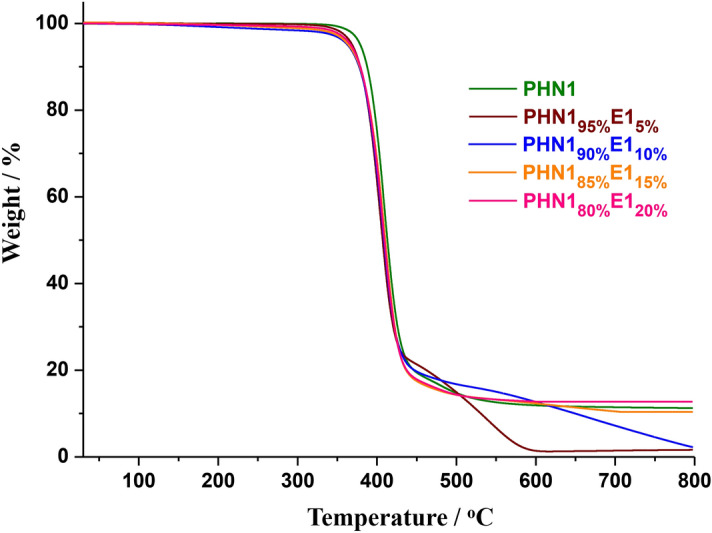
Thermogravimetric analysis (TGA) curves of PHN1_1−x_E1_x_ recorded from 25–800 °C at a heating rate of 10 °C/min under a nitrogen atmosphere.

**Figure 4:**
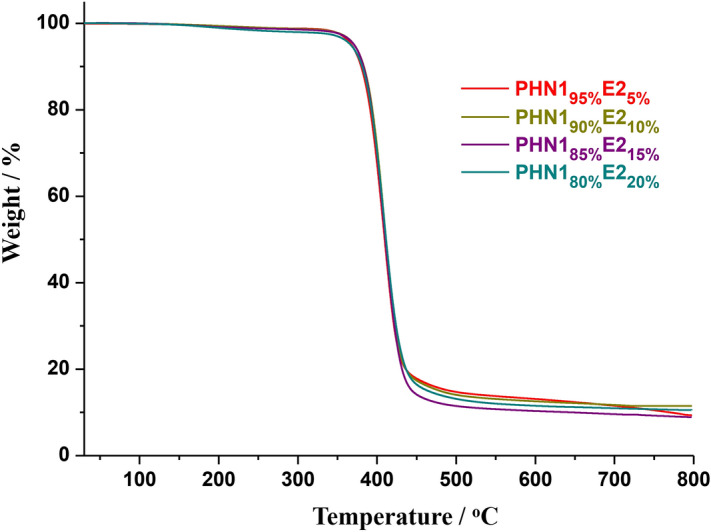
Thermogravimetric analysis (TGA) curves of PHN1_1−x_E2_x_ recorded from 25–800 °C at a heating rate of 10 °C/min under a nitrogen atmosphere.

Thermal stability is an important parameter of polymer materials in practical applications, and determines the long-term usability. In this study, thermal stabilities of the copoly(ether ester)s were investigated by thermogravimetric analysis (TGA). Weight-loss curves and the corresponding derivative curves were depicted in Fig. 3, Fig. 4 and Supplementary Fig. S18-S19, respectively. Thermal property parameters were summarized in Table 2 and the results suggested that PHN1_1−x_E1_x_ and PHN1_1−x_E2_x_ exhibited comparable thermal stability with the parent PHN1. Despite the content of eugenol-derived composition reached 20%, the temperature at which 5% weight loss (*T*_5%_) decreased just about 12 °C for both PHN1_1−x_E1_x_ and PHN1_1−x_E2_x_ relative to PHN1 (Table 2, entry 1, 5, and 9). Furthermore, PHN1_1−x_E1_x_ and PHN1_1−x_E2_x_ exhibited almost the same thermal stability regardless of the composition of copoly(ether ester)s. Based on the above results we conclude that the incorporation of eugenol-derived units had little influence on thermal stability of the final product. *T*_5%_ values were above 363 °C for all the samples, which is comparable with that of the petroleum-based poly(butylene terephthalate) (PBT) (*T*_5%_ = 371 °C) with appropriate molecular weights (*M*_n_ = 17,100, *Ð* = 2.4) reported by others^34^. The temperature for maximum degradation rate (*T*_d_) values were almost identical for both PHN1 and PHN1_1−x_E1(2)_x_, whose *T*_d_ values were fixed at about 410 °C and found to be insusceptible to the changes of content in eugenol-derived composition. Furthermore, PHN1_1−x_E1_x_ with eugenol-derived composition below or equal to 10% featured a two-step degradation mechanism. However, for PHN1_1−x_E1_x_ with eugenol-derived composition above 10% and the whole series of PHN1_1−x_E2_x_, single-step degradation was observed. This phenomenon could be explained by the mismatch of conformation of polymer chains at such specific compositions. Conclusion could be drawn from TGA analysis was that the copoly(ether ester)s featured excellent thermal stabilities, and the incorporation of eugenol-derived component actually had good compatibility with the parent PHN1. In a word, these bio-derived materials have excellent thermal stability.

**Table 2:** Thermal property parameters of the poly(ether ester)s.

Entry	Polymers	TGA			DSC
		*T*_5%_^a^ (°C)	*T*_d_^b^ (°C)	*W*^c^ (%)	*T*_g_^d^ (°C)
1	PHN1	379	409/480	11.3	13.1
2	PHN1_95%_E1_5%_	368	405/537	1.3	13.1
3	PHN1_90%_E1_10%_	363	407/612	2.2	7.7
4	PHN1_85%_E1_15%_	363	410	10.2	7.7
5	PHN1_80%_E1_20%_	367	408	12.8	4.8
6	PHN1_95%_E2_5%_	367	409	9.3	10.3
7	PHN1_90%_E2_10%_	371	408	11.4	7.5
8	PHN1_85%_E2_15%_	371	409	8. 8	5.0
9	PHN1_80%_E2_20%_	366	410	10.4	4.6
10	PBT^e^	371	408	2	31

^a^Temperature at which 5% weight loss.

^b^Temperature for maximum degradation rate.

^c^Remaining weight at 800 °C.

^d^Glass transition temperatures (*T*_g_) taken as the inflection points of the second heating DSC traces of precipitated samples at a heating/cooling rate of 10 °C/min.

^e^The data of poly(butylene terephthalate) (PBT) here was referenced from others^34^.

**Figure 5:**
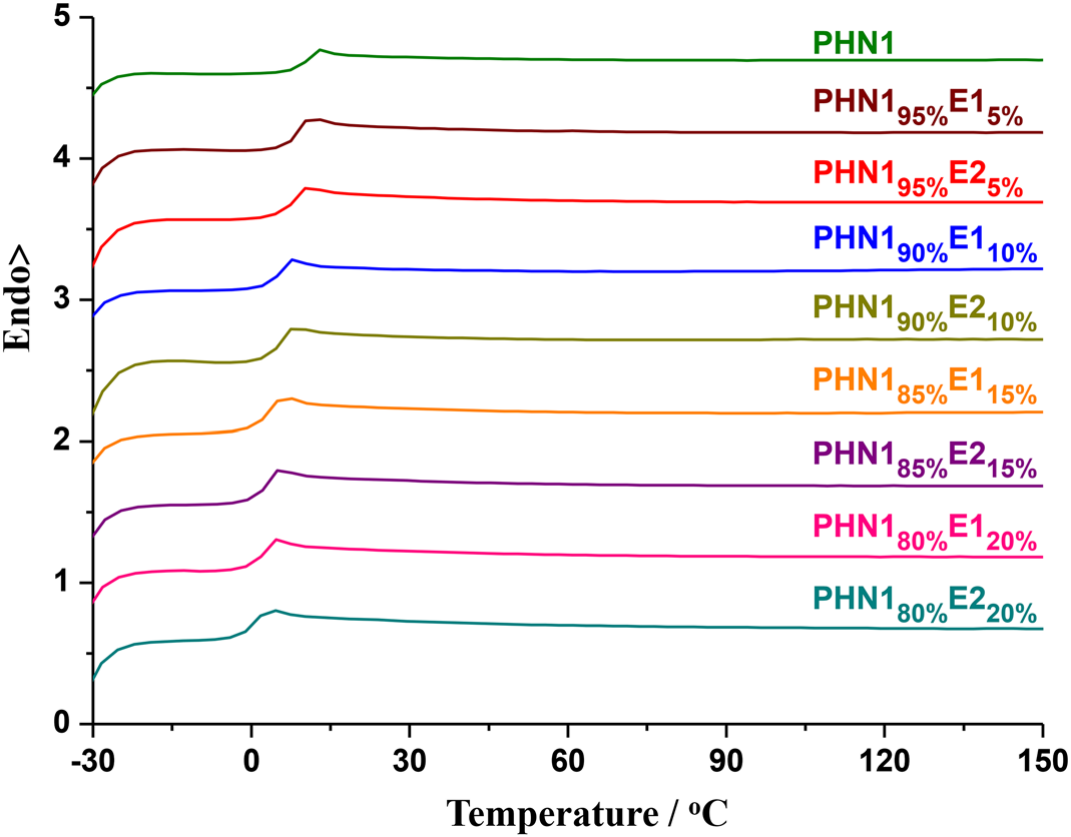
The second heating DSC curves for the poly(ether ester) samples carried out from − 30 to 150 °C at a heating/cooling rate of 10 °C/min.

Other thermal properties like glass transition (*T*_g_), melting and crystallization temperatures (*T*_m_, *T*_c_), together with their corresponding melting and crystallization enthalpies (*ΔH*_m_, *ΔH*_c_) are critical factors for the shape retention under normal practical use, as well as during molding process. Here these properties were studied by differential scanning calorimetric analysis (DSC). The DSC heating traces (2nd) are shown in Fig. 5, and the analytical data are gathered in Table 2.

Both PHN1_1−x_E1_x_ and PHN1_1−x_E2_x_ were found to feature obvious glass transition with *T*_g_ values ranging from 4.6 to 13.1 °C, which have a progressing decrease trend with the gradual increase of eugenol-based composition, thus caused many free volumes for segmental motion and low *T*_g_. PHN1_1−x_E1_x_ had a slightly higher *T*_g_ value than PHN1_1−x_E2_x_ when the content of eugenol-derived composition is the same, suggesting that PHN1_1−x_E2_x_ features a better flexibility than PHN1_1−x_E1_x_. The single glass transition in the second heating run for both two series of copoly(ether ester)s suggested that these samples could not crystallize from the melt during the first cooling run at the given cooling rate of 10 °C/min. The locally ordered packing of chain segments was not favoured. Although the *T*_g_ values gradually decreased with the incorporation of eugenol-derived units, the values changed not so much and were still in the scope of practical applications. Compared with our previous reported 1,10-decanediol-based copoly(ether ester)s (*T*_g_ <  − 8 °C)^26^, the *T*_g_ values (> 4.5 °C) of 1,6-hexanediol-based materials in this study has obvious improvement, which is beneficial for applying in a broad range of temperature.

### Powder X-ray diffraction analysis

**Figure 6:**
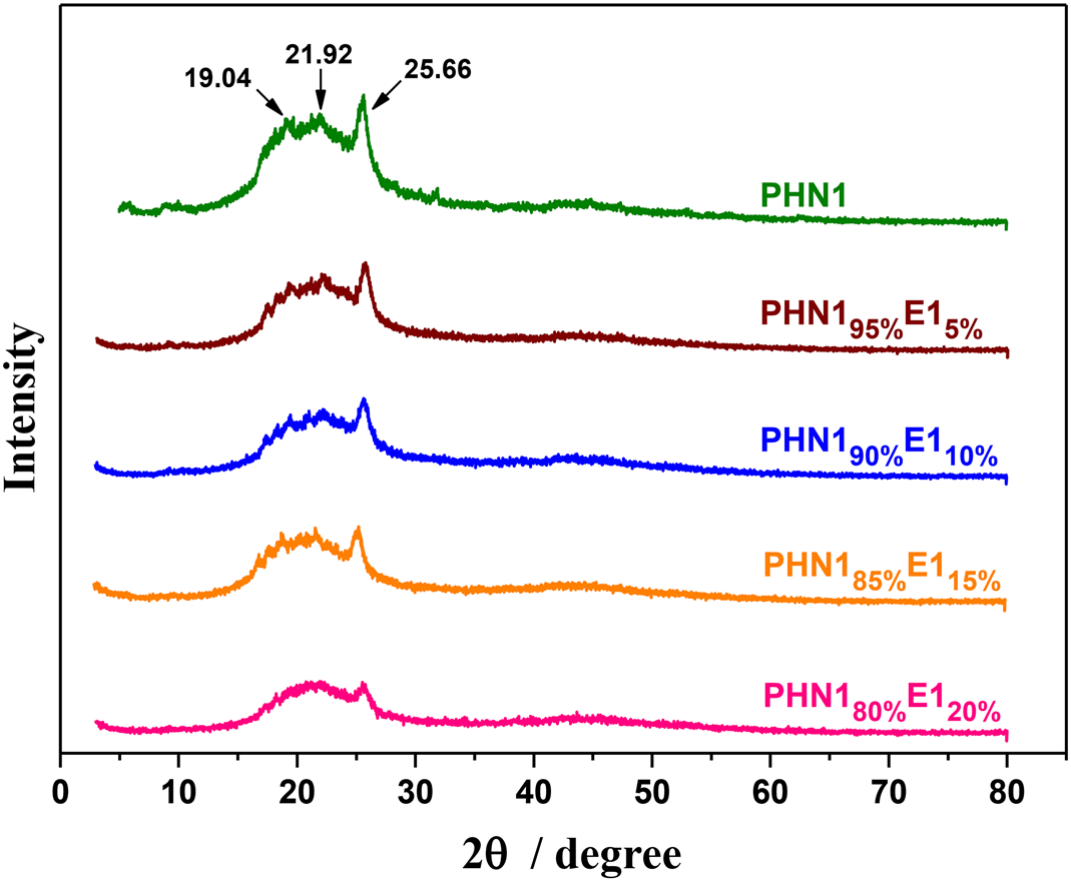
Powder WXRD profiles for PHN1 and PHN1_1−x_E1_x_.

In order to verify and reinforce the above DSC results and further study their crystalline ability of this kind of materials, powder wide-angle X-ray diffraction analysis (WXRD) was performed. The WXRD traces were depicted in Fig. 6 and Supplementary Fig. S20, and the diffraction data were collected in Table 3. All the samples were not able to form well discrete diffraction peaks characteristic of amorphous materials. The scattering pattern for PHN1 was featured by three reflections at 19.04°, 21.92°, and 25.66°, respectively, which corresponded to the triclinic crystal structure just like displayed by PBT^35,36^. Almost the same diffraction pattern was observed for all the poly(ether ester) materials when the diffraction angles and relative intensities were considered, indicating the crystalline mode of PHN1 was maintained in the copoly(ether ester)s. The crystallinity values can be calculated as the quotient between crystalline area and total area of the diffraction traces. The copoly(ether ester)s were found to be semi-crystalline and the crystallinities (*X*_c_) decreased with the increase of eugenol-derived composition. The discrepancy of material attribute from DSC and WXRD analysis could be ascribed to the difference of treatment method. The samples for WXRD were directly from precipitation in methanol, resulting in a certain degree of crystallization. The X-ray diffraction results confirmed that such materials still have slightly crystalline properties, rather than completely amorphous materials. The crystallinities gradually decrease with the insertion of eugenol-derived units.

**Table 3:** Powder X-ray diffraction data for the poly(ether ester)s.

Entry	Polymers	X-ray diffraction data			
		2θ (°)^a^			*X*_c_^b^
1	PHN1	19.04 m	21.92 m	25.66 s	0.22
2	PHN1_95%_E1_5%_	19.04 m	21.92 m	25.66 s	0.19
3	PHN1_90%_E1_10%_	19.04 m	21.92 m	25.66 s	0.18
4	PHN1_85%_E1_15%_	19.04 m	21.92 m	25.66 s	0.16
5	PHN1_80%_E1_20%_	19.04 w	21.92 w	25.66 m	0.15
6	PHN1_95%_E2_5%_	19.04 m	21.92 m	25.66 s	0.18
7	PHN1_90%_E2_10%_	19.04 m	21.92 m	25.66 s	0.16
8	PHN1_85%_E2_15%_	19.04 w	21.92 w	25.66 m	0.15
9	PHN1_80%_E2_20%_	19.04 w	21.92 w	25.66 m	0.13

^a^The diffraction angles measured in powder diffraction patterns for samples coming directly from precipitation in methanol and dried overnight. Intensities visually estimated as follows: *m* medium; *s* strong; *w* weak.

^b^Crystallinity index (*X*_c_) calculated as the quotient between crystalline area and total area. Crystalline and amorphous areas in the X-ray diffraction pattern were quantified using PeakFit v4.12 software.

### Stress–strain behaviour

**Figure 7:**
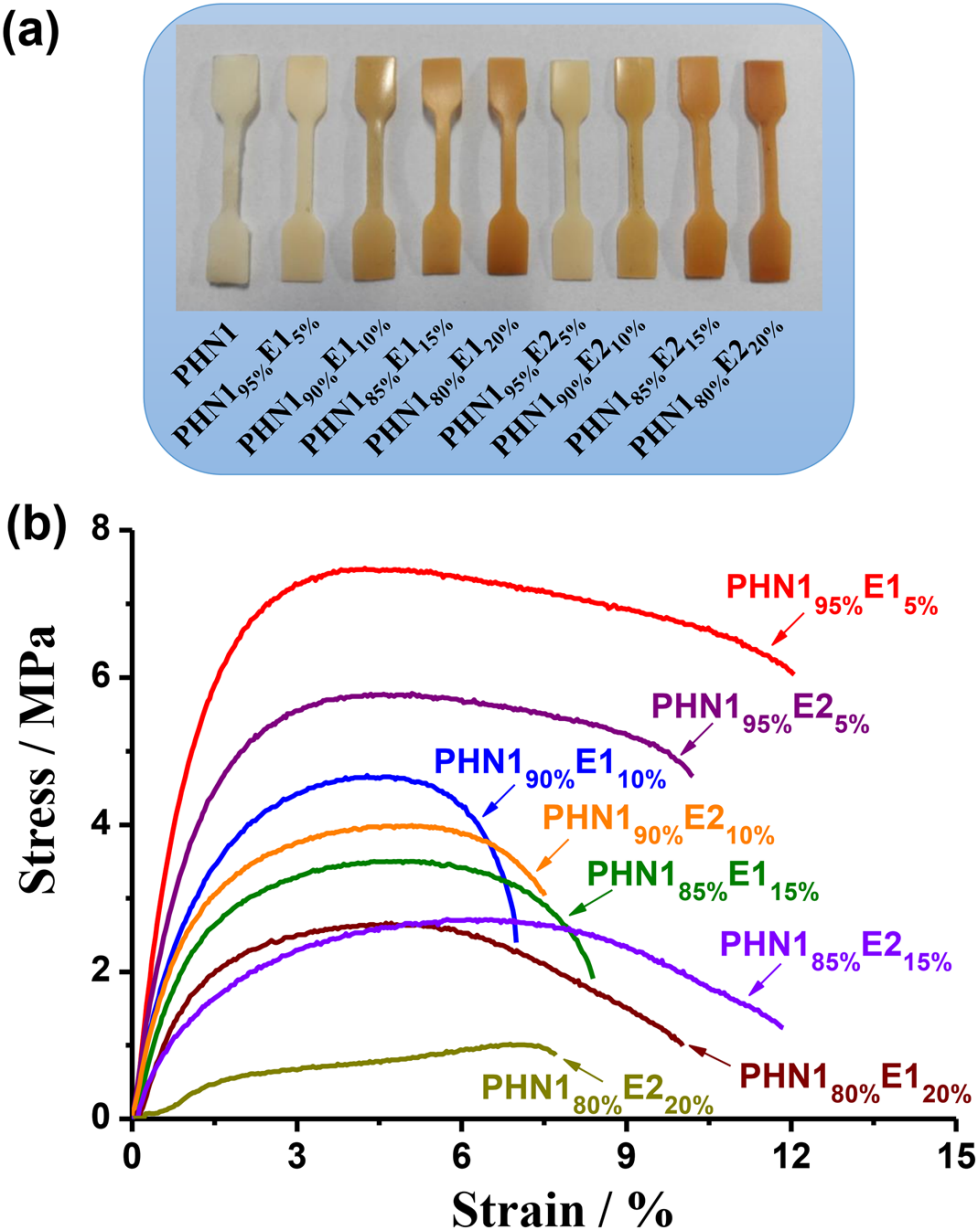
Stress–strain samples (**a**) and curves (**b**) of the poly(ether ester)s.

Mechanical properties (strength, toughness, ductility, etc.) are primary parameters to be considered for practical applications. To further study the synergistic effect of nipagin and eugenol-based components on mechanical properties, tensile assays of PHN1_1−x_E1_x_ and PHN1_1−x_E2_x_ copoly(ether ester)s were performed on dumb-bell shaped specimens (12 × 2 × 0.5 mm^3^), which were prepared by casting the chloroform solution (0.1 g/mL). The stress-stain curves were depicted in Fig. 7 and the mechanical property data were summarized in Table 4. Young’s modulus and tensile strength were found to decrease with the increase of eugenol-derived units in each series of samples. Furthermore, Young’s modulus and tensile strength for PHN1_1−x_E1_x_ was slightly higher than those of PHN1_1−x_E2_x_ when the content of eugenol-derived composition was the same. However, the elongations at break firstly decrease and then increase with the content of eugenol-derived composition increasing. For example, elongations at break decreased from 14.5% for PHN1 (the data of PHN1 here are original)^37^ to 7.0% for PHN1_90%_E1_10%_, and then increased to 10.0% for PHN1_80%_E1_20%_. The elongation at break didn't exhibit the same trends as the Young’s modulus and tensile strength do. This may be ascribed to the asymmetrical nature of eugenol-derived composition, leading to the difficult stacking and loose entanglement of polymer chains. Consequently, Young’s modulus and tensile strength decreases with the insertion of eugenol-derived composition, while the elongation at break did not feature the same variation tendency. Although PHN1 seems to feature the best mechanical properties (Young’s modulus, tensile strength and elongation) than those of copolymerized products, the necessity of copolymerization of nipagin and eugenol-derived monomers is also significant. Nipagin and eugenol are two structurally different building blocks, which will have significant influence on thermal (*T*_g_, *T*_5%_), crystalline (crystallinity), and mechanical properties (modulus, strength and ductility), which can be tailored for a broad range of applications, such as rubbery and elastomers, etc. depending on the application scenarios.

**Table 4:** Mechanical property parameters for the poly(ether ester)s.

Entry	Polymers	Mechanical properties^b^		
		Young’s modulus (MPa)	Tensile strength (MPa)	Elongation at break (%)
1	PHN1^a^	696 ± 24	17.2 ± 1.5	14.5 ± 2.2
2	PHN1_95%_E1_5%_	240.0 ± 14	7.5 ± 1.2	12.1 ± 4.2
3	PHN1_90%_E1_10%_	146.7 ± 12	4.7 ± 2.1	7.0 ± 3.6
4	PHN1_85%_E1_15%_	110.0 ± 16	3.5 ± 1.5	8.4 ± 5.0
5	PHN1_80%_E1_20%_	83.3 ± 11	2.7 ± 1.8	10.0 ± 4.5
6	PHN1_95%_E2_5%_	183.3 ± 28	5.8 ± 1.5	10.2 ± 2.4
7	PHN1_90%_E2_10%_	123.3 ± 25	4.0 ± 1.7	7.5 ± 3.2
8	PHN1_85%_E2_15%_	57.7 ± 12	2.7 ± 0.8	11.9 ± 4.8
9	PHN1_80%_E2_20%_	33.3 ± 8	1.0 ± 0.6	7.7 ± 4.6
10	PBT^c^	841 ± 15	42 ± 5	14 ± 3

^a^The data of PHN1 here has been reported in a previous report^36^.

^b^Young's modulus, tensile strength and elongation at break were calculated by averaging the data from three parallel test on dumb-bell shaped specimens (dimensions: 12 × 2 × 0.5 mm^3^) obtained from the casting of chloroform solutions at a concentration of 0.1 g/mL.

^c^The data of poly(butylene terephthalate) (PBT) here was referenced from others^34^.

When compared with the widely-used petroleum-based poly(butylene terephthalate) (PBT) (Table 4, entry 10)^34^, the polymer materials in this study presented inferior tensile strength and Young’s modulus. This was largely attributed to the insertion of eugenol-derived composition impeding the crystallization of copoly(ether ester)s significantly, together with tunable elongation at break. Although we initially targeted the substitutes of petroleum-based dimethyl terephthalate (DMT) and its derived PBT-like materials, the properties presented in this study were opposite. PBT are often used as food packaging materials, which require high *T*_g_, young modulus, and strength values, as well as specific barrier properties, but the polymer materials in this work are appropriate for other application scenarios (elastomer and rubbery), thanks to their modulated mechanical properties by insertion of disparate components. From the point of renewability, this is significant to broaden the types of polymer materials and application fields from bio-based starting materials. The harmony synergistic effect of nipagin and eugenol-derived composition could modulate the properties of such polymer materials in desired application fields.

## Conclusions

In order to prepare further sustainable and practical polymer materials from naturally occurring biomass, renewable nipagin and eugenol-based aromatic copoly(ether ester) materials were synthesized via the melt polycondensation method. The effect of introduction of eugenol-derived units on thermal, crystalline, and mechanical properties was emphatically studied. TGA results revealed that the copoly(ether ester)s featured fairly high thermal stability despite the content of eugenol-derived composition reached 20%. DSC and WXRD data suggested the amorphous nature for both homopoly(ether ester) and copoly(ether ester)s, regardless of the content of highly asymmetrical eugenol-based composition. Young’s modulus and tensile strengths were also found a decreasing tendency with the insertion of eugenol-based composition. More importantly, elongation at break can be tunable via the different content of eugenol-based composition. The cooperative interaction of renewable nipagin and eugenol-derived building blocks provides a practical and suitable way to obtain polymeric materials with desired performance and suitable for various application scenarios.

## Materials and methods

### Reagents and materials

Nipagin (99%, Aladdin, Shanghai, China), eugenol (99%, Aladdin), methyl thioglycolate (99%, Aladdin), methyl chloroacetate (98%, Aladdin), 2,2-dimethoxy-2-phenylacetophenone (DMPA) (99%, Aladdin) and tetrabutyl titanate (TBT) (99.5%, Aladdin), 1,4-dibromobutane (98%, *J*&*K* Scientific, Beijing, China), and 1,6-hexanediol (98%, *J*&*K* Scientific) were used as received. All solvents (Tianjin Chemical Reagent Co., Ltd, Tianjin, China) were analytical grade and used as received without further purification. Silica-gel slices used for thin-layer chromatography (TLC) were purchased from Qingdao Haiyang Chemical Co. Ltd (Qingdao, China).

### General instrumentation

^1^H NMR and ^13^C NMR spectra were recorded in CDCl_3_ at 25 °C on a Bruker AVANCE III NMR spectrometer operating at 400 MHz and 100.6 MHz, respectively. Fourier transform infrared spectra (FTIR) were recorded on a Bio-Rad FTS6000 spectrophotometer at 25 °C. Polymer samples were prepared by grinding the polymeric materials adequately with KBr powder, followed by compressing the mixture to form a pellet. The molecular weights and polydispersity (*Ð*) values of the samples were determined by gel permeation chromatography (GPC, Waters 2414 differential refraction detector) at 35 °C. Tetrahydrofuran (THF) was used as the eluent at a flow rate of 1.0 mL/min. The average molecular weights were calibrated against monodisperse polystyrene (PS) standards. Thermogravimetric analysis (TGA) was carried out using a NETZSCH TG209 instrument. In a typical run, polymer sample was heated from 25 to 800 °C under a nitrogen atmosphere at a rate of 10 °C/min. The temperature leading to 5% weight loss (*T*_5%_), temperature for maximum degradation rate (*T*_max_), and residue weight (%) at 800 °C were recorded. Differential scanning calorimetric (DSC) analysis was carried out by a Mettler-Toledo DSC Q100 calorimeter from TA Instruments. Polymer sample after drying was first heated from room temperature to 150 °C and hold at this temperature for 10 min to eliminate thermal history, then cooled to − 30 °C. Glass transition temperature (*T*_g_) was observed from the second heating run. All runs were carried out at a rate of 10 °C min^−1^. Indium was used as the calibration standards for temperature. Wide X-ray diffraction (WXRD) patterns were recorded on a D/max-2500 diffractometer using CuKα radiation with a wavelength of 0.1542 nm for dried powder samples. Crystallinity (*X*_c_) was calculated as the quotient between crystalline area and total area. Crystalline and amorphous areas in the X-ray diffraction pattern were quantified by the PeakFit v4.12 software (SeaSolve Software Inc.) with multi-functions (automatic baseline correction (Linear, D2), deduction of reductant peaks, AutoFit (Savitzky-Golay smoothing, Gauss amplification), AutoScan, etc.). Tensile assays were performed at a stretching rate of 50 mm/min at 25 °C on a Testometric AX Universal Strength Testing Machine.

### Preparation of nipagin and eugenol-based copoly(ether ester)s

PHN1 homopoly(ether ester), PHN1_1−x_E1_x_, and PHN1_1−x_E2_x_ (x = 0%, 5%, 10%, 15%, 20%) copoly(ether ester)s were prepared from the mixtures of N1, E1 or E2, and 1,6-hexanediol with the pre-calculated stoichiometric composition. The polymerization was performed in a 25 mL Schleck round-bottom flask equipped with a nitrogen inlet, a vacuum distillation outlet, and a magnetic stirrer bar. The polymerization scheme is illustrated in Fig. 1. A slight excess molar ratio of diesters to diol (1:1.05) was employed to ensure complete polymerization of ester groups and hydroxyl termination. Tetrabutyl titanate (TBT, 0.6% molar relative to diester) was used as the catalyst. Before transesterification, the system was purged with nitrogen for 15 min to ensure no oxygen remaining and to avoid being oxidized during polymerization. Transesterification was carried out under a low nitrogen flow at 140–180 °C for 3–5 h. Polycondensation was then carried out under a 0.03–0.06 mbar vacuum at 180–200 °C for 3–5 h until stirrer bar was stuck, suggesting the completion of polymerization. The reaction mixture was cooled down to room temperature, while normal pressure was recovered with nitrogen to prevent degradation of product. The crude product was dissolved in a minimum amount of chloroform and precipitated in an excess of methanol to remove oligomers and the excess diols. Final product was collected by filtration, thoroughly washed with methanol, and dried in vacuo overnight.

### PHN1 homopoly(ether ester)

^1^H NMR (400 MHz, 25 °C, CDCl_3_): *δ* = 7.99–7.97 (m, 2H, Ar–*H*), 6.91–6.89 (m, 2H, Ar–*H*), 4.66 (s, 2H, -O-C*H*_2_-CO-), 4.27–4.17 (m, 4H, -COO-C*H*_2_-), 1.76–1.62 (m, 4H,-COOCH_2_-C*H*_2_-), 1.49–1.25 (m, 4H, -COOCH_2_CH_2_-C*H*_2_-) ppm; ^13^C NMR (100.6 MHz, 25 °C, CDCl_3_): *δ* = 168.02 (-OCH_2_-*C*O-), 165.68 (Ar-*C*O-), 161.13 (Ar–*C*), 131.23 (Ar–*C*), 123.47 (Ar–*C*), 113.91 (Ar–*C*), 64.98–64.78 (m, -O-*C*H_2_-CO-), 64.34–64.23 (m, -COO-*C*H_2_-), 28.34–28.00 (m, -OCH_2_-*C*H_2_-), 25.45–24.98 (m, -OCH_2_CH_2_-*C*H_2_-) ppm; FTIR: 2949, 1781 (C=O), 1711 (C=O), 1606, 1510, 1253, 1170, 1105, 977, 848, 770, 697 cm^−1^.

### PHN1_1−x_E1_x_ copoly(ether ester)s

^1^H NMR (400 MHz, 25 °C, CDCl_3_): *δ* = 7.95–7.93 {m, (1-x)∙2H; Ar–*H*}, 6.88–6.86 {m, (1-x)∙2H; Ar–*H*}, 6.72–6.68 (m, x∙2H; Ar–*H*), 6.63–6.61 (m, x∙H; Ar–*H*), 4.63 {s, (1-x)∙2H; ArO-C*H*_2_-CO-}, 4.60 (s, x∙2H; ArO-C*H*_2_-CO-), 4.30–4.09 {m, (1-x)∙4H; -COO-C*H*_2_-}, 4.15–4.02 (m, x∙4H; -COO-C*H*_2_-), 3.81 (s, x∙3H; ArO-C*H*_3_), 3.16 (s, x∙2H; -S-C*H*_2_-CO-), 2.62–2.57 (m, x∙4H; Ar-C*H*_2_-CH_2_-C*H*_2_-S-), 1.88–1.81 {q, ^3^*J*(H,H) = 8.0 Hz, x∙2H; ArCH_2_-C*H*_2_-CH_2_S-}, 1.79–1.65 {m, (1-x)∙2H; ArCOOCH_2_-C*H*_2_-}, 1.65–1.52 {m, (1-x)∙2H + x∙4H; -COOCH_2_-C*H*_2_-}, 1.50–1.17 {m, (1-x)∙4H + x∙4H; -COOCH_2_CH_2_-C*H*_2_-} ppm; ^13^C NMR (100.6 MHz, 25 °C, CDCl_3_): *δ* = 170.48 (-SCH_2_-*C*O-), 169.18 (ArOCH_2_-*C*O-), 168.29 (ArOCH_2_-*C*O-), 166.02 (Ar-*C*O-), 161.38 (Ar–*C*), 149.54 (Ar–*C*), 145.59 (Ar–*C*), 135.74 (Ar–*C*), 131.51 (Ar–*C*), 123.82 (Ar–*C*), 120.23 (Ar–*C*), 114.63 (Ar–*C*), 114.17 (Ar–*C*), 112.60 (Ar–*C*), 66.68 and 65.11 (ArO-*C*H_2_-CO-), 65.38–64.50 (-COO-*C*H_2_-), 55.87 (ArO-*C*H_3_), 34.15 (-S-*C*H_2_-CO-), 33.59 (Ar-*C*H_2_-CH_2_CH_2_S-), 31.99 (-S-*C*H_2_-CH_2_CH_2_Ar), 30.50 (ArCH_2_-*C*H_2_-SCH_2_-), 28.67–28.54 (m, -COOCH_2_-*C*H_2_-), 28.40–28.27 (m, -COOCH_2_CH_2_-*C*H_2_-) ppm; FTIR: 2934, 2860, 1756 (C = O), 1703 (C = O), 1603, 1510, 1422, 1265, 1166, 1105, 1070, 974, 846, 765, 693 cm^−1^.

### PHN1_1−x_E2_x_ copoly(ether ester)s

^1^H NMR (400 MHz, 25 °C, CDCl_3_): *δ* = 7.92–7.90 {m, (1-x)∙2H; Ar–*H*}, 6.85–6.83 {m, (1-x)∙2H; Ar–*H*}, 6.73–6.71 (m, x∙H; Ar–*H*), 6.63–6.59 (m, x∙2H; Ar–*H*), 4.59 {s, (1-x)∙2H; ArO-C*H*_2_-CO-}, 4.19–4.10 {m, (1-x)∙4H; -COO-C*H*_2_-}, 4.03–3.97 {m, (1-x)∙4H + x∙4H; ArO-C*H*_2_-CH_2_- and -COO-C*H*_2_-}, 3.75 (s, x∙6H; ArO-C*H*_3_), 3.13 (s, x∙4H; -S-C*H*_2_-CO-), 2.67–2.46 (m, x∙8H; Ar-C*H*_2_-CH_2_-C*H*_2_-S-), 2.02–1.88 (m, x∙4H; ArOCH_2_-C*H*_2_-), 1.88–1.76 {q, ^3^*J*(H,H) = 8.0 Hz, x∙4H; ArCH_2_-C*H*_2_-CH_2_S-}, 1.75–1.63 {m, (1-x)∙2H; ArCOOCH_2_-C*H*_2_-}, 1.62–1.47 {m, (1-x)∙2H + x∙4H; -COOCH_2_-C*H*_2_-), 1.46–1.12 {m, (1-x)∙4H + x∙4H; -COOCH_2_CH_2_-C*H*_2_-} ppm; ^13^C NMR (100.6 MHz, 25 °C, CDCl_3_): *δ* = 170.29 (-SCH_2_-*C*O-), 168.09 (ArOCH_2_-*C*O-), 165.78 (Ar-*C*O-), 161.22 (Ar–*C*), 149.20 (Ar–*C*), 146.57 (Ar–*C*), 133.87 (Ar–*C*), 131.31 (Ar–*C*), 123.62 (Ar–*C*), 120.13 (Ar–*C*), 114.00 (Ar–*C*), 113.21 (Ar–*C*), 112.20 (Ar–*C*), 68.58 (ArO-*C*H_2_-CH_2_-), 64.90 (ArO-*C*H_2_-CO-), 64.42–64.31 (-COO-*C*H_2_-), 55.71 (ArO-*C*H_3_), 33.95 (-S-*C*H_2_-CO-), 33.39 (Ar-*C*H_2_-CH_2_CH_2_S-), 31.81 (-S-*C*H_2_-CH_2_CH_2_Ar), 30.42 (ArCH_2_-*C*H_2_-SCH_2_-), 28.43–28.15 (m, -COOCH_2_-*C*H_2_-), 25.84 (ArOCH_2_-*C*H_2_-), 25.53–25.06 (m, -COOCH_2_CH_2_-*C*H_2_-) ppm; FTIR: 2935, 2860, 1756 (C=O), 1708 (C=O), 1603, 1510, 1459, 1264, 1166, 1107, 1071, 976, 847, 809, 765, 693 cm^−1^.

## Supplementary Information

Supplementary Information.
