# Supplementary material for: Synergistic interaction of renewable nipagin and eugenol for aromatic copoly(ether ester) materials with desired performance
Source: Sci Rep. 2021 Dec 16;11:24119. doi: 10.1038/s41598-021-03614-z (PMC8677751; doi:10.1038/s41598-021-03614-z)
Supplement: Supplementary file 1 — Supplementary Information. [file 41598_2021_3614_MOESM1_ESM.pdf]

Supplementary information for

Synergistic interaction of renewable nipagin and eugenol for aromatic copoly(ether ester) materials with desired performance

Keling Hu[1]* Huachao Sui[2] & Dongping Zhao[2]

[1] School of Materials Science and Engineering, Tiangong University,
Tianjin 300387, P. R. China.

[2] School and Hospital of Stomatology, Tianjin Medical University,
Tianjin 300070, P. R. China.

Correspondence to *hukeling@tiangong.edu.cn*

1. Experimental Section

Synthesis of Nipagin and Eugenol-derived Dimethyl Esters

The preparation methods for nipagin and eugenol-derived dimethyl esters **N1**, **E1** and **E2** have been reported previously (Figure S1-S8).[1,2]

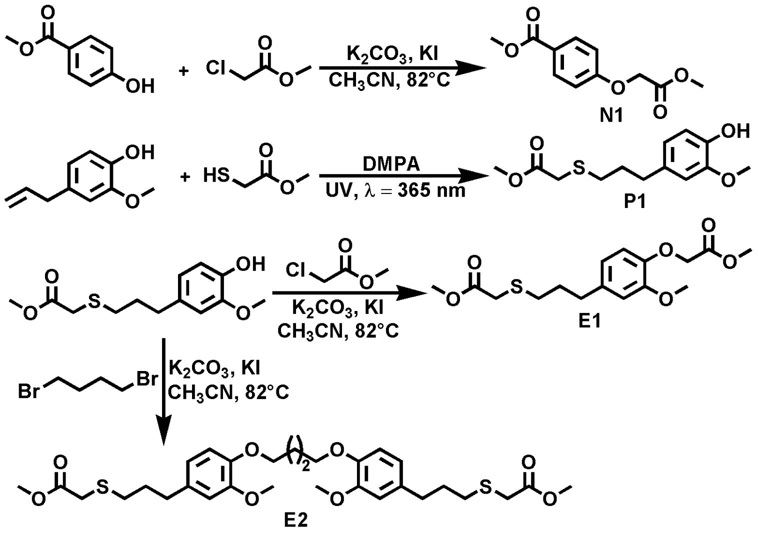

Figure S1. Synthetic routes for the preparation of nipagin and eugenol-derived dimethyl esters **N1**, **E1** and **E2**.

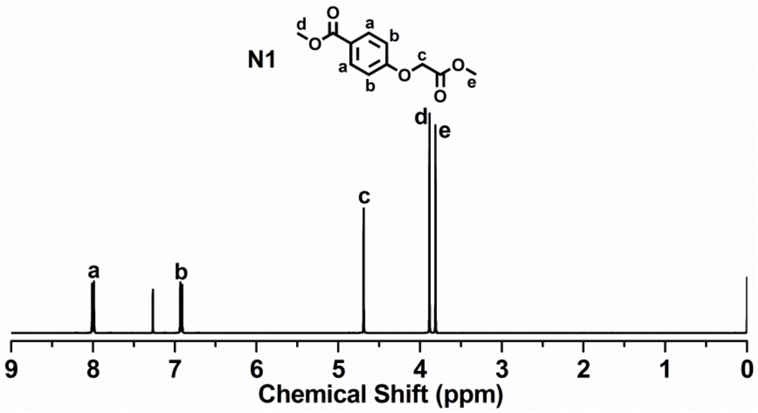

Figure S2. ^{1}H NMR spectrum of **N1**.

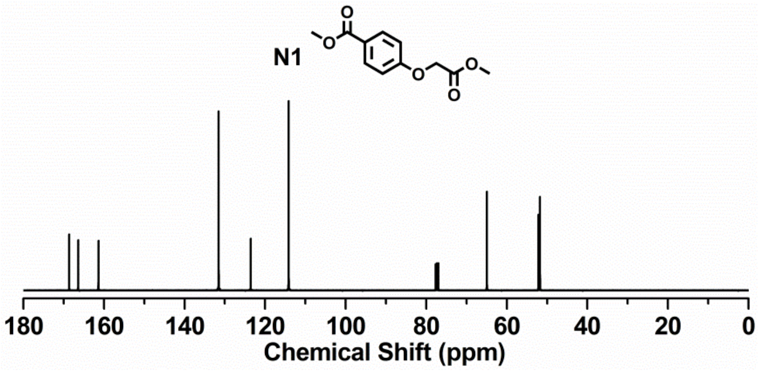

Figure S3. ^{13}C NMR spectrum of **N1**.

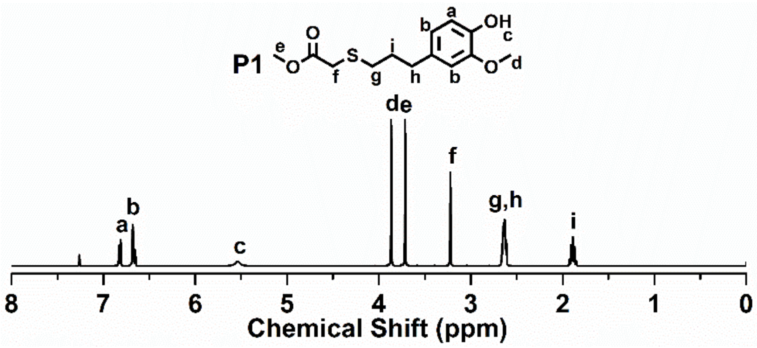

Figure S4. ^{1}H NMR spectrum of **P1**.

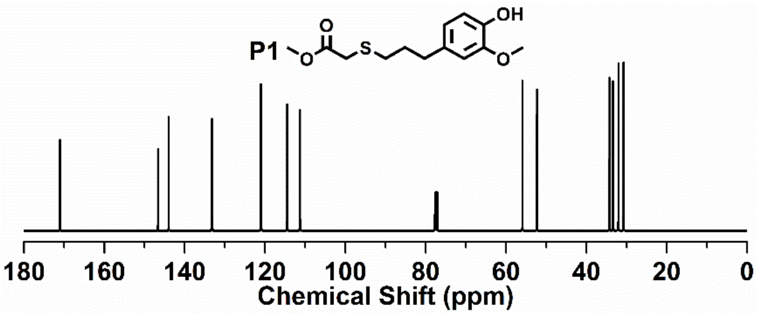

Figure S5. ^{13}C NMR spectrum of **P1**.

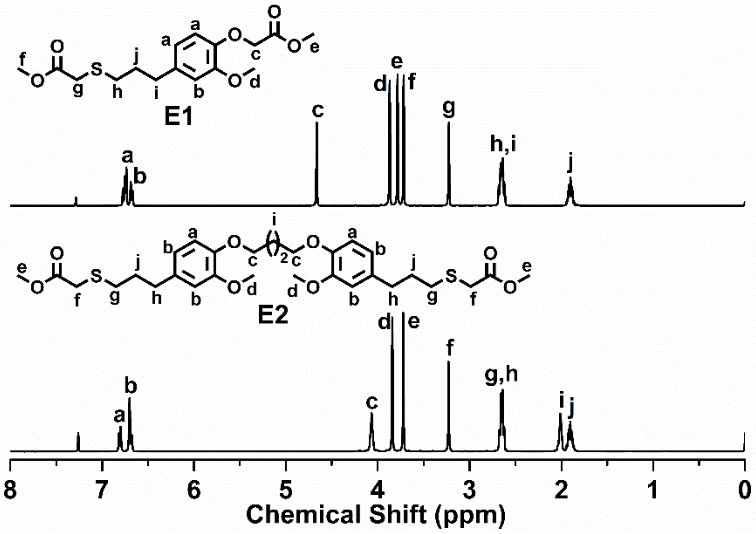

Figure S6. ^{1}H NMR spectra of **E1** and **E2**.

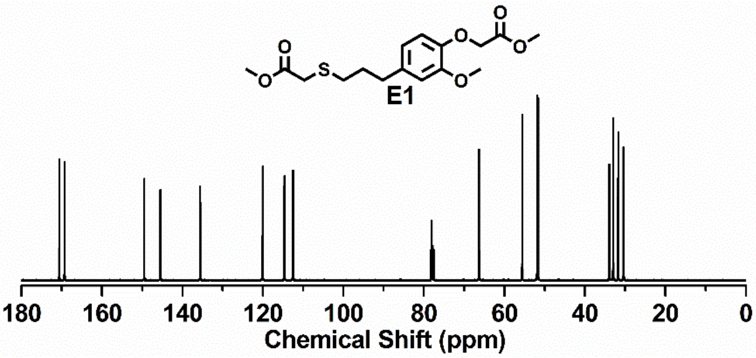

Figure S7. ^{13}C NMR spectrum of **E1**.

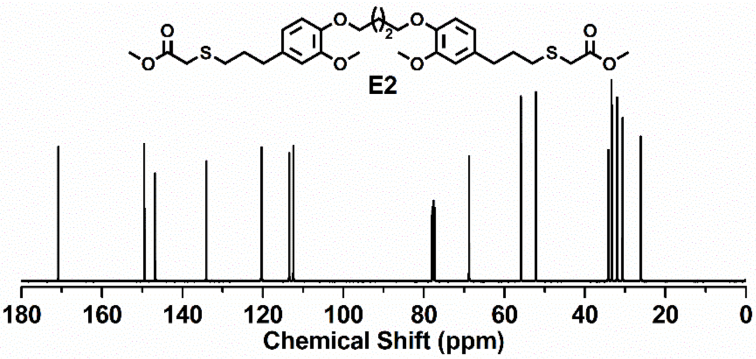

Figure S8. ^{13}C NMR spectrum of **E2**.

2. Experimental Results

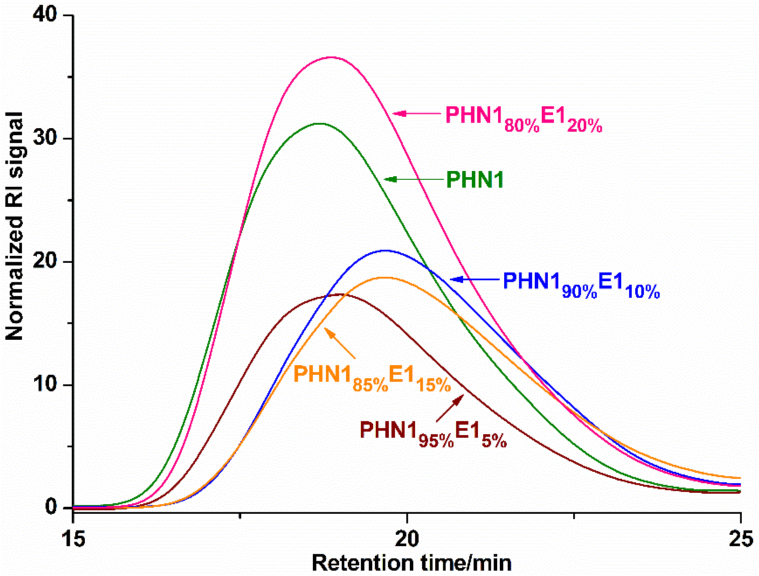

Figure S9. SEC traces of **PHN1$_{1-x}$E1$_x$** copoly(ether ester)s.

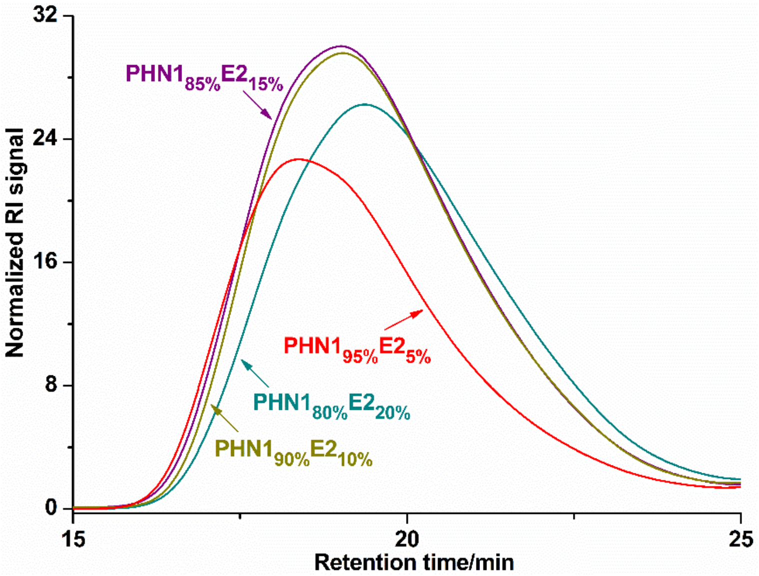

Figure S10. SEC traces of **PHN1$_{1-x}$E2$_x$** copoly(ether ester)s.

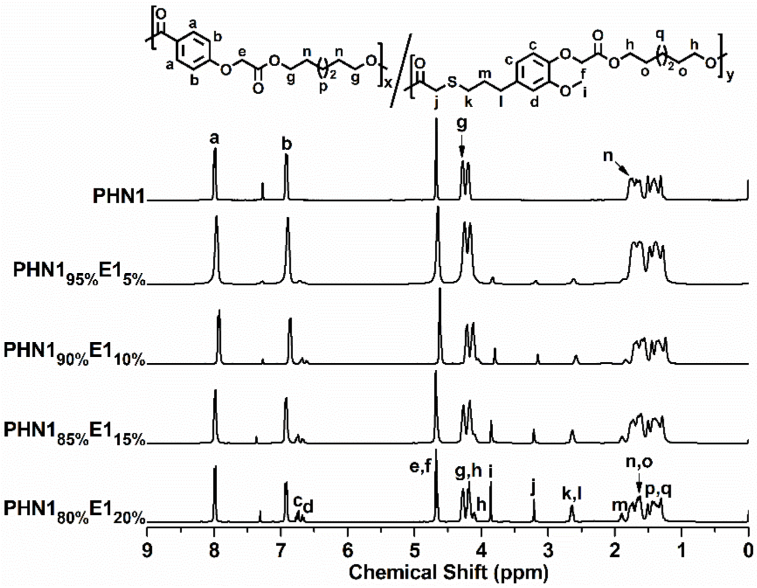

Figure 11. ^{1}H NMR spectra of **PHN1$_{1\text{-}x}$E1$_x$** copoly(ether ester)s.

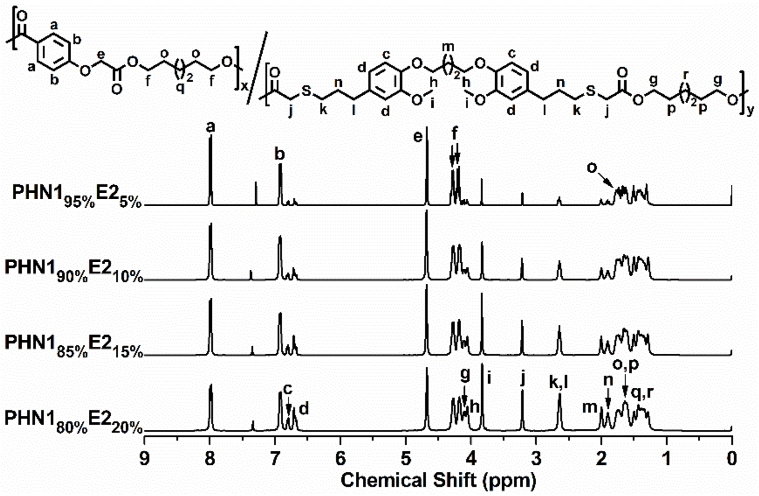

Figure S12. ^{1}H NMR spectra of **PHN1$_{1\text{-}x}$E2$_x$** copoly(ether ester)s.

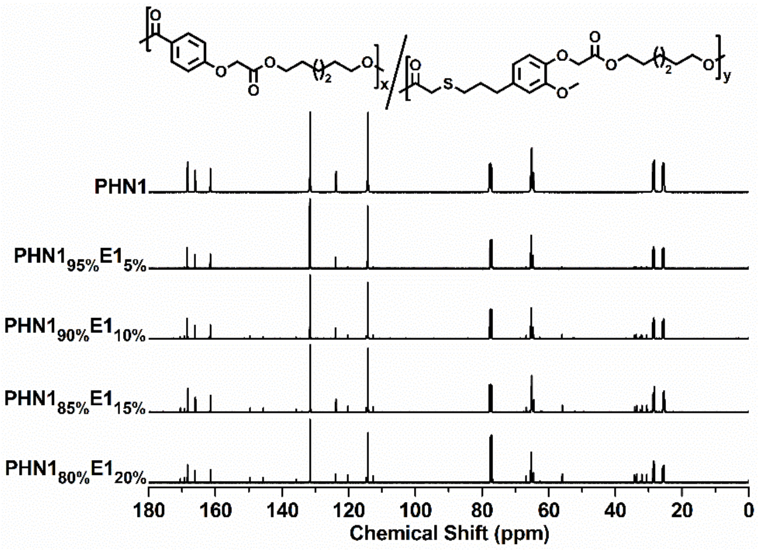

Figure S13. ^{13}C NMR spectra of **PHN1$_{1-x}$E1$_x$** copoly(ether ester)s.

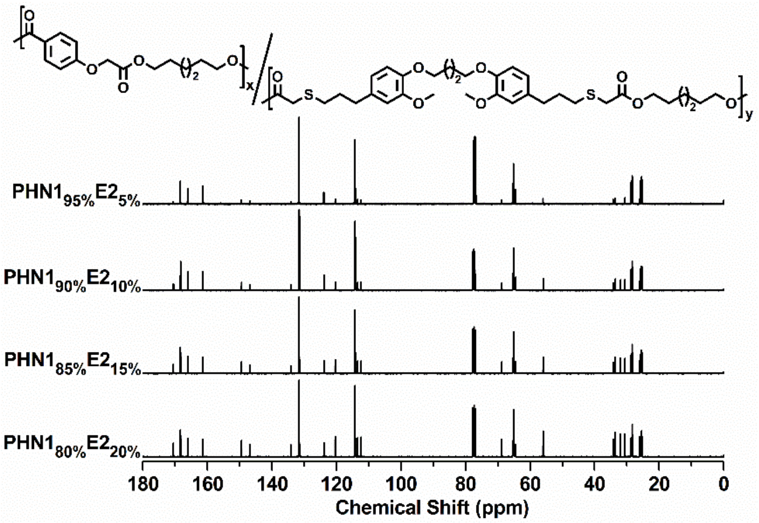

Figure S14. ^{13}C NMR spectra of **PHN1$_{1-x}$E2$_x$** copoly(ether ester)s.

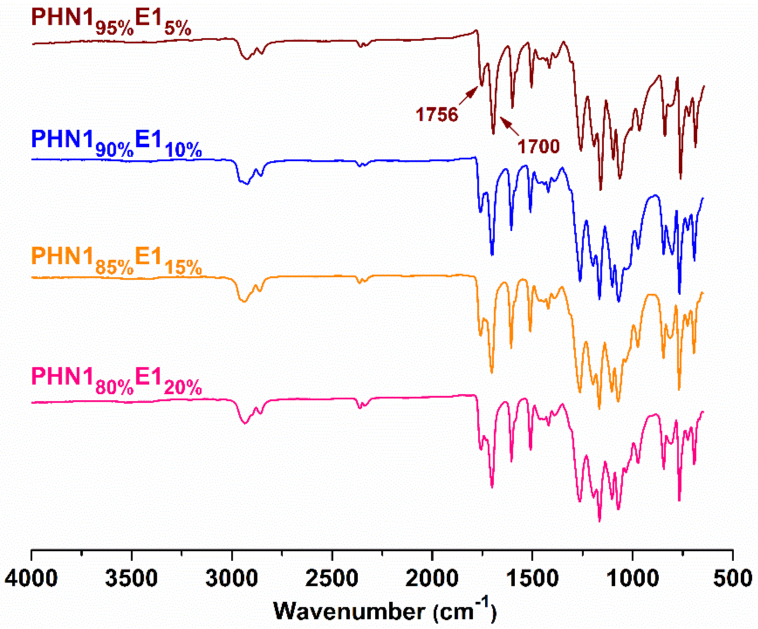

Figure S15. FTIR spectra of **PHN1$_{1-x}$E1$_x$** copoly(ether ester)s.

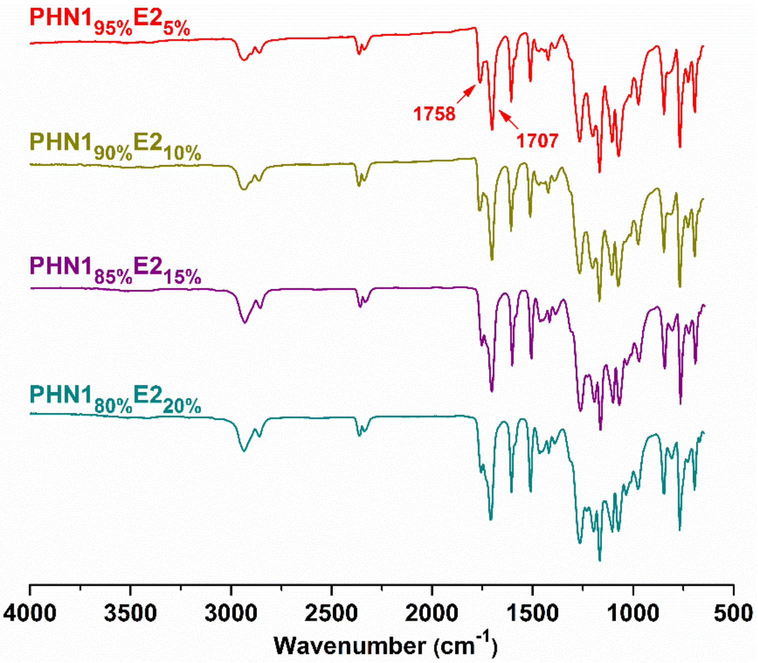

Figure S16. FTIR spectra of **PHN1$_{1-x}$E2$_x$** copoly(ether ester)s.

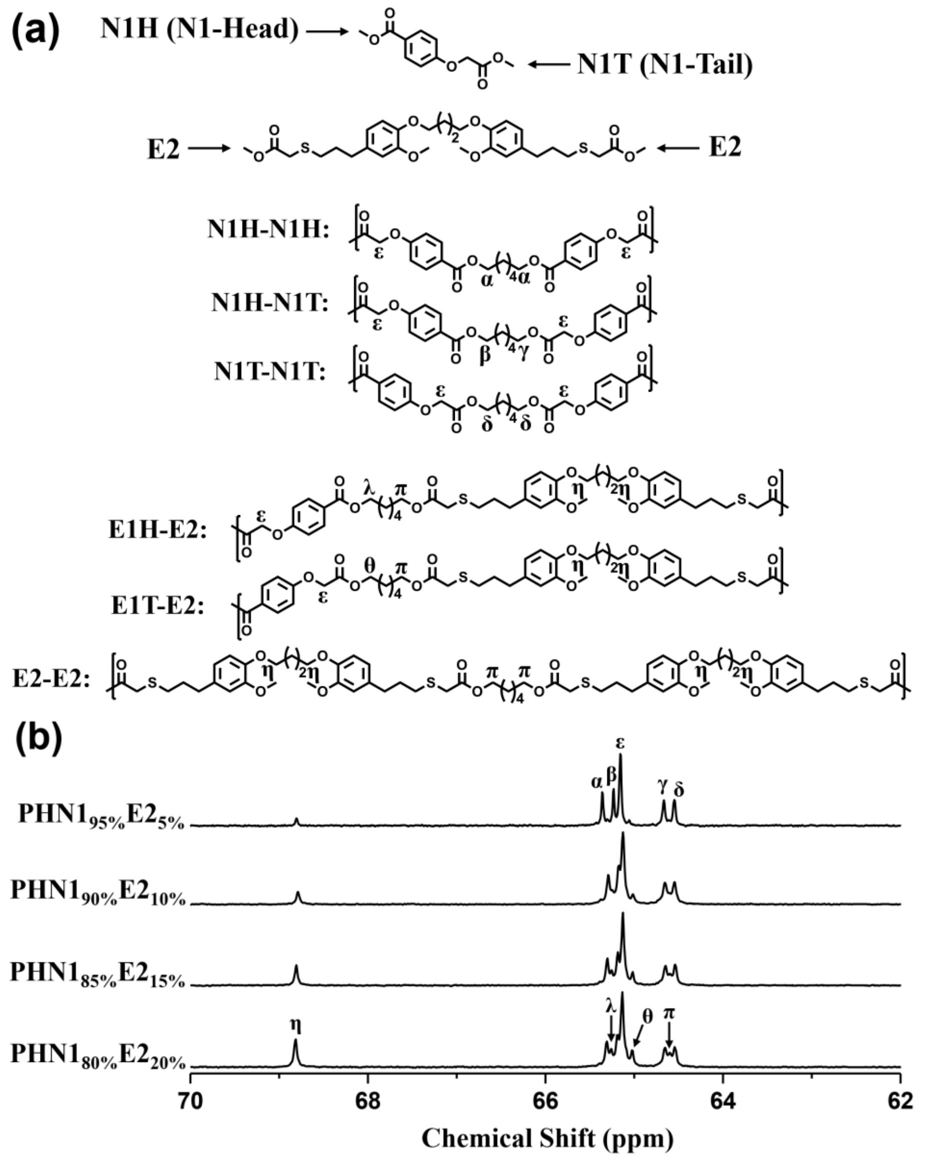

Figure S17. The splitting situations of different methylene carbons under different magnetic environment for PHN1$_{1-x}$E2$_x$ with the indications of dyads to which they are assigned. The symbols (α, β, γ, δ, ε, η, θ, λ, and π) represent the different carbon atoms, their attribution and splitting mode in ^{13}C NMR are indicated in the Figure S17a and S17b, respectively. The ^{13}C NMR spectra were recorded in $CDCl_3$ at 25 °C on a Bruker AVANCE III NMR spectrometer operating at 100.6 MHz.

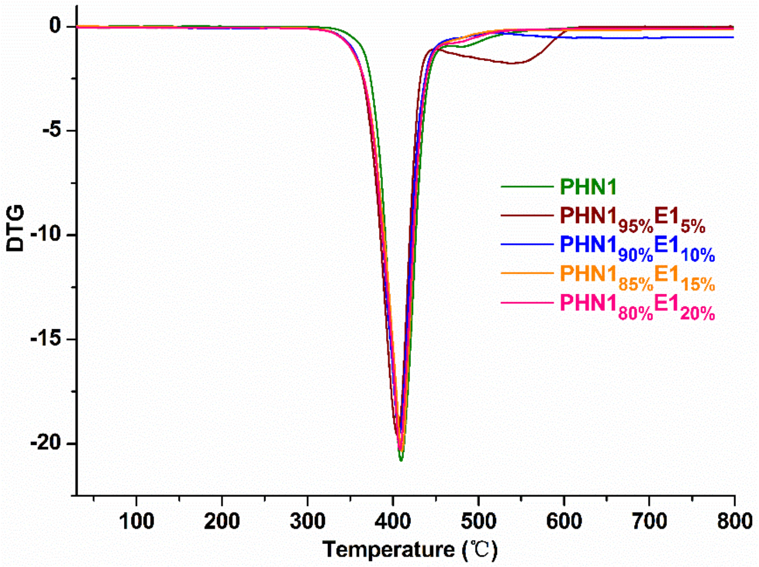

Figure S18. TGA derivative curves of **PHN1$_{1\text{-}x}$E1$_x$** copoly(ether ester)s.

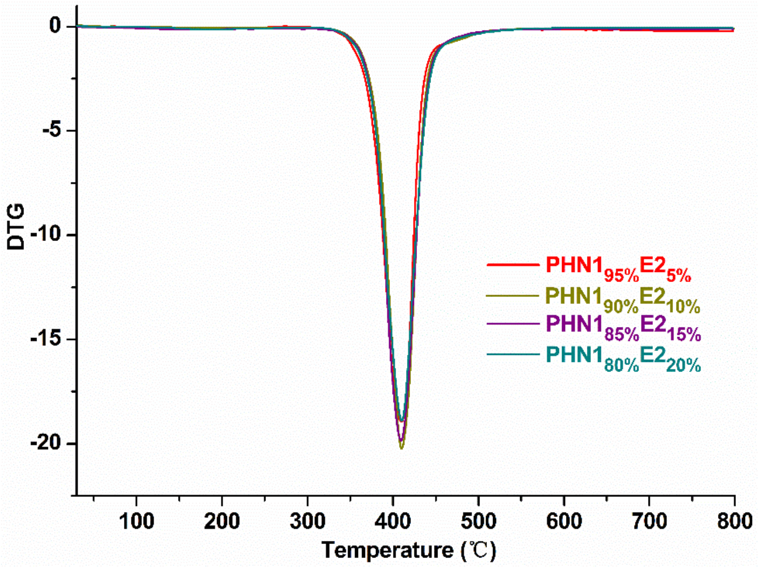

Figure S19. TGA derivative curves of **PHN1$_{1\text{-}x}$E2$_x$** copoly(ether ester)s.

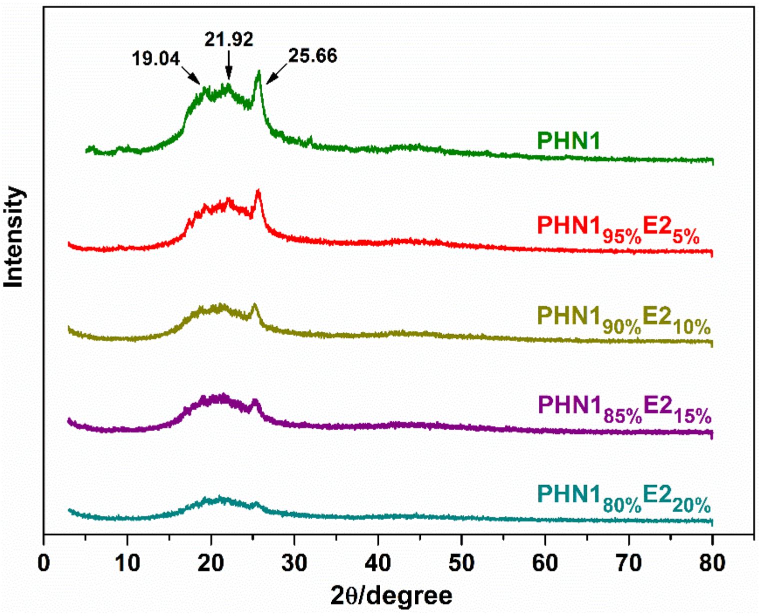

Figure S20. Powder WXRD profiles for **PHN1$_{1-x}$E2$_x$** copoly(ether ester)s.

3. References

1. Hu, K., Zhao, D., Wu, G. & Ma, J. *Polym. Chem.* **6**, 7138-7148 (2015).

2. Hu, K., Zhao, D., Wu, G. & Ma, J. *J. Polym. Sci., Part A: Polym. Chem.* **54**, 2171-2183 (2016).
